# Fuzzy Control of Pressure in a Water Supply Network Based on Neural Network System Modeling and IoT Measurements

**DOI:** 10.3390/s22239130

**Published:** 2022-11-24

**Authors:** José Vinicius Santos de Araújo, Juan Moises Mauricio Villanueva, Marcio Miranda Cordula, Altamar Alencar Cardoso, Heber Pimentel Gomes

**Affiliations:** 1 Renewable and Alternatives Energies Center (CEAR), Electrical Engineering Department (DEE), Campus I, Federal University of Paraiba (UFPB), Joao Pessoa 58051-900, Brazil; 2 Water and Sewerage Company of Paraíba CAGEPA, Joao Pessoa 58015-901, Brazil; 3 Technology Center (CT), Department of Civil and Environmental Engineering (DECA), Campus I, Federal University of Paraiba (UFPB), Joao Pessoa 58051-900, Brazil

**Keywords:** fuzzy control system, neural network models, IoT module ESP8266, Scada-LTS, control pressure

## Abstract

As hydroenergetic losses are inherent to water supply systems, they are a frequent issue which water utilities deal with every day. The control of network pressure is essential to reducing these losses, providing a quality supply to consumers, saving electricity and preserving piping from excess pressure. However, to obtain these benefits, it is necessary to overcome some difficulties such as sensing the pressure of geographically distant consumer units and developing a control logic that is capable of making use of the data from these sensors and, at the same time, a good solution in terms of cost benefit. Therefore, this work has the purpose of developing a pressure monitoring and control system for water supply networks, using the ESP8266 microcontroller to collect data from pressure sensors for the integrated ScadaLTS supervisory system via the REST API. The modeling of the plant was developed using artificial neural networks together with fuzzy pressure control, both designed using the Python language. The proposed method was tested by considering a pumping station and two reference units located in the city of João Pessoa, Brazil, in which there was an excess of pressure in the supply network and low performance from the old controls, during the night period from 12:00 a.m. to 6:00 a.m. The field results estimated 2.9% energy saving in relation to the previous form of control and a guarantee that the pressure in the network was at a healthy level.

## 1. Introduction

The hydro-energy losses inherent to water distribution systems (WDSs) are a highly relevant theme due to the large amount of water consumed for pumping and the high water losses [1], especially considering the context of water scarcity and high electricity costs [2]. According to the 2021 SNIS report, the water losses in distribution (i.e., the difference between the volume produced and the volume consumed) in Brazil were approximately 40.1%. In the northeast, the losses reached an average of 46.3%. Likewise, countries are aligned with the Sustainable Development Goals, following the guidelines of Goal 6: Clean water and sanitation. This goal promotes good hygiene to help eliminate neglected tropical diseases.

In line with the high system losses, the increase in size and complexity of water distribution networks require great technical, organizational, and financial efforts from sanitation companies [3]. Thus, sanitation companies have been under strong pressure to reduce expenses and increase the quality of the service provided, especially in Brazil after the sanction of the new Legal Framework for Basic Sanitation (a Brazilian law), which will require adequate structural conditions and quality standards for the service provided [4].

In this scenario, actions aimed at minimizing the losses related to inefficient water transport management are fundamental and urgent, especially in the application and modernization of control and monitoring systems capable of optimizing operational decisions [5]. Implementation and expansion of supervisory, control, and data acquisition (SCADA) systems, low-cost Internet of Things (IoT) technologies, system modeling via artificial intelligence (AI), and modern controls are some of the actions that can provide greater operational efficiency.

According to [6], pressure control is fundamental for reducing real water losses in a WDS because it is the main factor that influences the number of leaks and the flow of these leaks. In addition, adequate pressure control logic reduces the system’s energy consumption, since the pump sets do not need to operate continuously at full load due to the variable dynamics of water consumption throughout the day. Still, there is also the challenge of implementing control logic with good performance and simple tuning due to the great nonlinearities of the WDS and the complex interconnections between the pipes, which hinders the use of some types of classical control, such as proportional–integral–derivative (PID) control.

Several engineering solutions have been proposed to increase the energy efficiency of a WDS, such as pressure control in a given area through pressure-reducing valves (PRVs), turbines, pumps as turbines, and variable speed pumps (VSPs). However, PRVs are designed to produce load losses and do not guarantee adequate energy efficiency strategies. On the other hand, control strategies based on PID control allow for adjusting pressures properly as long as the systems are linear, which is one of the main limitations of this type of controller. In this way, controllers based on fuzzy logic arise to deal with nonlinear systems, with acceptable performance even with disturbances and variable demands in the water supply networks [7].

In this context, another big challenge to controlling hydrostatic pressure in water supply networks refers to the distance between the sensors (level relays, water flow meters, hydrostatic probes, and others) and the process actuators (pump sets, frequency converters, and valves), which makes it difficult to relate them physically via cabling. Often, a water lift station is more than 3 km away from one of its influence zones. Thus, using industrial Ethernet protocols and low-cost IoT technologies is an excellent alternative for linking the elements of the water supply system, extending the network’s monitoring and facilitating the realization of control.

Given these considerations, this work aims to propose a low-cost monitoring and control solution for a water supply network by using ScadaLTS as a supervisory system, ESP8266, and hydrostatic pressure sensors to collect data from the hydraulic network and fuzzy logic developed in Python for control in order to ensure adequate pressure for consumers and the health of the pipes.

To reduce the risks of applying the proposed control methodology directly to the hydraulic network, it was also proposed to model the hydraulic network using artificial neural networks (ANN) so that the tuning of the fuzzy controller would be performed earlier in this model. Thus, this work aims to present an efficient and simplified method, using low-cost resources for monitoring (ESP8266) and efficient control logic in an easy-to-learn programming environment (Python) that water utilities can replicate without difficulty.

The main contributions of the proposed work are summarized as follows: (1) more efficient pressure control of the water supply network, (2) decreasing the number of pipe rupture occurrences due to excessive pressure in the network, and (3) saving energy by using frequency converters as actuators in a closed-loop system.

The text is structured in sections to facilitate reading, each detailing a specific part. Section 2 will present the state of the art of similar research. Section 3 gives a brief explanation of some concepts such as the WDS, frequency converters, SCADA systems, process modeling, and control systems. Subsequently, in Section 4, details about the case study plant and the software and hardware resources used are given. Section 5 discusses the results obtained for modeling the hydraulic network and the fuzzy controller’s performance. Finally, Section 6 gives a review of the results obtained, exploring the contributions and bottlenecks for future studies.

## 2. Related Works

The National Electric Energy Agency (ANEEL, Brazil) estimated that Brazilian industries consumed 35.83% of the electricity produced in 2017. Knowing that water utilities fall into this category, in that same year, these industries were responsible for 11.3 TWh. In [8], the authors stated that motor-pump sets are responsible for approximately 80–90% of the electrical consumption of pumping stations. In this scenario, it becomes a challenge for water supply networks to develop techniques that seek to improve hydropower efficiency continuously.

In [9], strategies are defined to improve the energy efficiency of WDSs, considering the improvement in the hydraulic efficiency of the pumps and electrical efficiency of the motors and using frequency converters, increasing the efficiency of the systems while taking into account the variable demand of the water distribution system. Another approach consists of controlling the pressure in the water distribution system with significant operational improvements, such as increasing the use life of the pipes, preventing ruptures due to excess pressure, increasing the reliability of the water supply to the population, reducing hydraulic transients, reducing water consumption, and the reduction of water leaks. Pressure control strategies are also essential in scenarios with regions with irregular topographies, especially in regions with low and high zones, where the need to increase pressure to meet the high zones leads to excessive pressure in the low zone, causing the rupture of pipes.

With regard to the control actions found in the literature to solve the problem of pressure control in water supply networks, there is [10], where the authors simulated control of the PID family to two pressure-reducing valves to control the minimum and maximum pressures of an area in Barcelona. Their simulations indicated that reducing the pressure in the network could decrease leakage losses in that area by about 3.65%, saving 11 m${}^{3}$ of water per day.

In [11,12,13], the authors introduced pressure control strategies using current automation tools, such as the use of a programmable logic controller (PLC) and frequency converters as well as fuzzy and fuzzy-PID control strategies, ensuring pressure stability in the supply network. In this work, dynamic response comparisons were performed, showing that the fuzzy-PID controller presented fast responses, with low overshoot and high noise immunity, but it required the construction of a very accurate knowledge base from the specialist.

In [14], the use of an adaptive neural network for optimal maintenance of the head of the pumping system studied was proposed, eliminating excess pressure in 36 critical points. For this, it was proposed to regulate the pump speed as an actuator to control the pressure in the network. Through tests on an experimental bench, the authors trained a neural network to develop an adaptive neural controller using the Levenberg–Marquardt algorithm, presenting a low maximum error in the steady state (maximum of 1.502%) and following the reference signal, even though these varied over time. Thus, real-time pressure controllers based on artificial neural networks (ANNs) are viable technical options that can be implemented to increase the system’s energy and hydraulic efficiency.

In [15], a new artificial neural network (ANN) controller was developed to improve the operation of water distribution systems (WDSs), including in its algorithm the specific energy consumption as a parameter. The technique was applied to control the pressures in an experimental configuration that emulated a WDS with two consumption zones with different topographies. Real-time control performance was proven based on the dynamic performance, steady state performance, and specific power consumption. The experimental results had good performance when following the set points. They provided a reduction in the specific energy consumption between 15.1% and 17.8% concerning the uncontrolled system and savings that ranged from 2.5% to 8.1% over the ANN’s performance based on pressure control alone. Through this methodology, it was possible to increase the energy efficiency of the pumping systems by adjusting the opening or closure of a valve and the rotation speeds of a conventional pump and a booster pump in real-time.

In [7], the actions of three topologies of modern controllers were compared: the fuzzy, neural, and neuro-fuzzy controllers. The authors also compared the energy efficiency and the average pressure of the system throughout the day, showing great performance for the neural and neuro-fuzzy controllers and reducing energy consumption by up to 79.3% at certain times of the day compared with a system without a control. Therefore, combined pressure control strategies are being used as control strategies implemented on the shop floor of water supply network automation.

In [16], the use of a fuzzy derivative controller to control the pressure of a 1200-mm pipeline by manipulating the opening angle of a valve using open source supervisory systems (ScadaBR) to maintain the pressure between 76 and 84 mH_2_O was proposed, managing to automate the process that an operator previously performed. In this work, the importance of low-cost automation implementations was shown, which allowed the monitoring and control of supply networks, increasing the energy and hydraulic efficiency of the system.

Because of the recent relevant works in the area, it was observed that there were developments for controllers based on artificial intelligence techniques. Thus, the authors of this paper have proposed the development of a fuzzy controller for pressure control in a water supply network, considering the good performance of fuzzy control applied in nonlinear systems found in the literature [17,18,19].

In addition, with the advent of technology, free software-based supervisory systems have been adopted in water utilities as a tool for low-cost automation solutions, such as for supplying water, as a tool for low-cost automation solutions, such as SCADA-BR and SCADA-LTS, among others. This software, for WDSs, is essential for monitoring the operational status of the hydraulic network and quickly detect leaks, motor failures, and pressure quality in networks.

In this scenario, intelligent control strategies are applied to water supply systems to optimize operational efficiency, equalize pressures in networks, and introduce energy efficiency actions. However, there are challenges to be overcome, mainly concerning the lack of registrations on the network, lack of modeling, and the presence of nonlinear systems.

## 3. Background Definitions

In this section, the theoretical foundation of the elements and resources that are explored in this work will be laid out, starting by defining a water supply system, its components, its operation, and the main difficulties inherent in water supply networks and describing some of the solutions that research has proposed to optimize the efficiency of the network.

Subsequently, the elements of the monitoring system that are being used as a tool to fulfill one of the stages of the work will be explored, describing the ScadaLTS supervisory system, ESP8266 development board for IoT applications, the Modbus remote terminal unit (RTU), and Modbus transmission control protocol (TCP) communication protocols as well as the HyperText Transfer Protocol (HTTP) internet protocol.

Then, we will address more directly the artificial neural networks and their ability to learn patterns of behavior through data, and finally, we will address the control systems in general, the characteristics of the main types of control, and the most used one in water supply networks, specifically fuzzy control, and its components.

### 3.1. Water Supply System

The water supply system is composed of structures, equipment, and devices that aim to provide water with adequate quality, quantity, and regularity so that it meets the demands of users according to the standards required for consumption [20].

**Figure 1:**
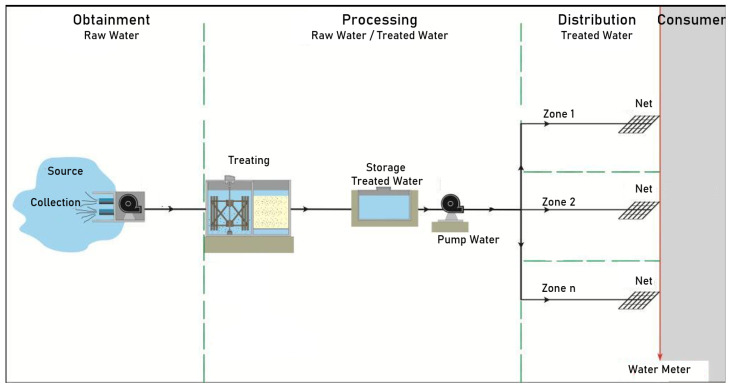
Details of operation of a water system.

To deliver water to consumers, water transportation goes through three stages, namely obtaining, processing, and distribution. In obtaining, the raw water is taken and transported from springs to the processing units, consisting of specific units for the capturing, lifting, and adduction of raw water. In the processing units, the water is treated to meet the requirements of the consumer (household, industrial, commercial, etc.), consisting of units for the treatment, storing, lifting, and adduction of treated water, and finally, the distribution sector is responsible for delivering the treated water to consumers [20]. Figure 1 illustrates these three stages.

Focusing on the treated water distribution stage, according to [20], the elements that compose the water distribution sector are comprised of the treated water pumping station (TWPS), the supported reservoir, the elevated reservoir, and the water distribution network.

The supported reservoir is usually underground and, as its name suggests, has the function of supporting the distribution system, acting as an extra reserve of treated water, and it essential at times when the volume of water required by the demand network is greater than the inflow volume.

In the TWPSs, the pumps are set to pump water from the supported reservoir to the elevated reservoir, which supplies the consumer units. However, in some situations, the water is pumped directly to the consumer units, requiring a more rigorous hydro-energetic performance evaluation.

Thus, part of the success and efficiency of the water distribution system is the knowledge of the characteristics of the distribution network and the motor-pump sets, which must be equalized according to the pressures established by the ABNT NBR 12218/1994 standard that requires a minimum network pressure of at least 10 mH_2_O and a maximum of 50 mH_2_O.

### 3.2. Frequency Converters

One of the strategies for controlling the rotation speed of the motor pump and, consequently, controlling the volume of water injected into the network is using frequency converters. These devices, aside from controlling the rotation speed of the motors, come with some on-board utilities for acting as a protection device for problems such as a lack of balance between phases, overload, and voltage drop, among others, also enabling motor control by torque, providing a soft start, and reducing reactive power consumption through harmonic filtering [21].

According to [22], the use of frequency converters can avoid waste by improving the system’s operational control. According to [3], frequency converters have become unanimously implemented. Due to the ability of AC drives to control the power injected into the pump sets and balance the power factor supplied by the grid through efficient rectifier circuits, alternating current (AC) drives are also synonymous with savings in electricity consumption.

Thus, many researchers have studied the use of AC drives to control pressure in the supply network, with many having an emphasis on energy efficiency, such as those in [3,14,23]. There are still works studying the use of converters for sewage lift stations, such as in [24].

### 3.3. Monitoring and Control Systems

A supervisory system is an essential element for obtaining the productive efficiency of an industrial process, allowing monitoring and tracking through data collection performed by data acquisition devices of information from physical facilities (also referred to as plants) [25].

Alternatively known as SCADA systems, the supervisory systems, employing computing and communication technologies, can group data collected from various field equipment, even if they are geographically dispersed, and present them in a friendly way to the operator through graphical resources and multimedia content.

**Figure 2:**
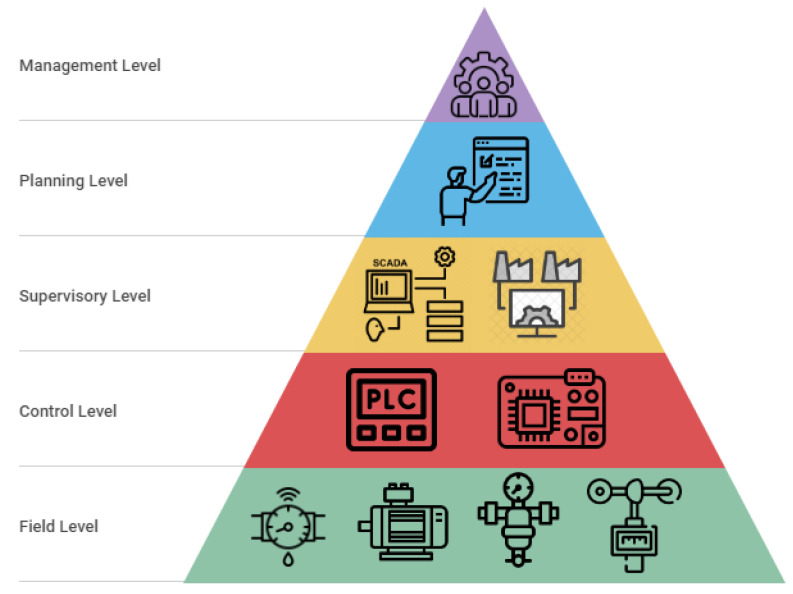
Automation pyramid. Source: author.

In this way, the monitoring and supervision system is composed of three basic elements: the SCADA works as the brain of the system by grouping, analyzing, and acting upon the collected data and information, the acquisition devices are responsible for extracting the data from the field equipment and sending these data to the SCADA system, and finally, the field equipment measures and acts upon the process variables. De maneira resumida, neste trabalho, pode-se considerar que seu impacto ocorre principalmente nos 3 níveis inferiores da pirâmide da automação, a qual o SCADA pertence ao nível que interliga as aplicações de campo com o lado da gestão e planejamento dos recursos, conforme illustrated in Figure 2.

### 3.4. System Modeling

One of the surest ways to optimize the operation of a real industrial process is by performing simulations of a virtual model of the plant. With proper modeling, it is possible to analyze the behavior of a system when subjected to a set of inputs that, when applied to the model, produces a set of outputs.

Modeling, therefore, reduces the chances that a physical industrial process behaves unexpectedly when subjected to the same inputs previously simulated, which provides greater reliability for an application to be implemented and operate successfully.

One of the computational tools used for system modeling is the artificial neural network (ANN). Modeling using ANNs is more efficient than other modeling techniques when the mathematical modeling of the system is complex and nonlinear, with hard modeling using differential equations, transfer functions, or the state space. Usually, this limitation is evidenced in nonlinear and time-varying systems, as in hydraulic processes, and extensive supply networks with many variables [26,27].

### 3.5. Control System

Several scientific and engineering works have applied classical PID controllers on pressure-reducing valves to control water supply networks with acceptable performances, reducing pressures in the networks, reducing leakages, and saving energy. On the other hand, techniques based on fuzzy, neuro-fuzzy, and adaptive control have been used as current tools, especially in systems where it is not possible to build a mathematical model or highly nonlinear or time-varying systems. These control techniques have been widely used, and in this work, a fuzzy pressure control system for a water supply network will be developed.

## 4. Materials and Methods

This section will recapitulate the problem of supply in a water distribution network, taking as an example a case study carried out in a water pumping station and its consumer units to describe the elements that compose it, the system constraints, and the desired performance goal.

Once the control of consumer unit pressure through the regulation of the rotation speed of a frequency inverter was implemented, control routines control the level of support and the level of compensation through the regulation of the opening angle of butterfly-type electric valves.

In addition, we will describe all the elements present in the case study, whether they are equipment, computational tools, or control routines, and the role of each one in the search for efficient an water supply.

### 4.1. Problem Description: Real Water Pumping Station

A common problem in a water supply network refers to the regulation of the pressure reaching the consumer units for two main reasons:

(1) Some water supply companies have not yet modernized their monitoring systems, so many times, one does not have the data (at least digitized data) about the current pressure in the consumer units, not having an adequate parameter to evaluate the quality of the supply.
(2) When the pressure data are available, there is still another obstacle, that being the distance between the WPS and the consumer units being reasonably high (in kilometers in general), which makes it difficult to measure the pressure located in the consumer units for a controller located in the WPS by wired means.

This way, many times the control is accomplished by an operator who operates on the on and off states of the motor-pump sets with the compensation level as a reference, acting to keep the water tank level high and, consequently, having enough head to supply the population but with the commitment to avoid overflowing the water tank.

However, another restriction for the system refers to the level of the support reservoirs, because the motor-pump sets cannot operate with them at a low levels due to the entry of air in the pump’s suction being harmful to its use life. In this way, controlling the arrival of water to the supports is also fundamental.

**Figure 3:**
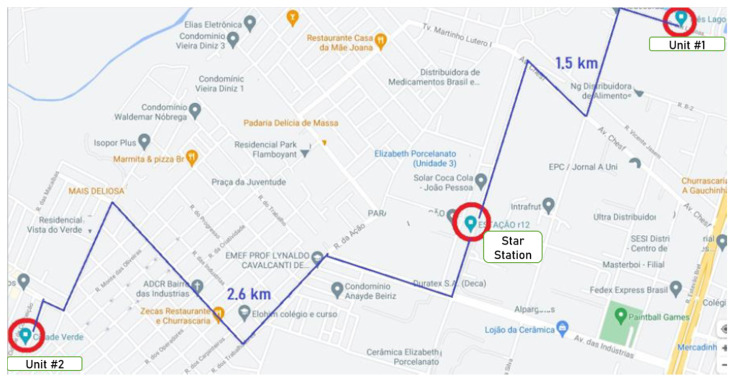
Map of the water supply network. Adapted from Google Maps (2021).

Such problems are present in the case study’s WPS, which we will refer to as the Star WPS. This pumping station supplies a region of the city with influence areas at distances greater than 2.5 km, as seen in Figure 3. Its main problem is during the night period, when the demand for water drops strongly, causing rapid growth of pressure in the network and, consequently, making the pipes rupture frequently.

This WSS is composed of equipment for sensing, acting, and controlling the network, which are as follows:

(1) Two frequency converters (pump station);
(2) One compensation tank (pump station);
(3) Two motor-pump sets (pump station);
(4) Two valves (pump station);
(5) Two supported tanks (pump station);
(6) Three pressure transducers (one in the pump station and two in the consumer units);
(7) One Siemens controller.

**Figure 4:**
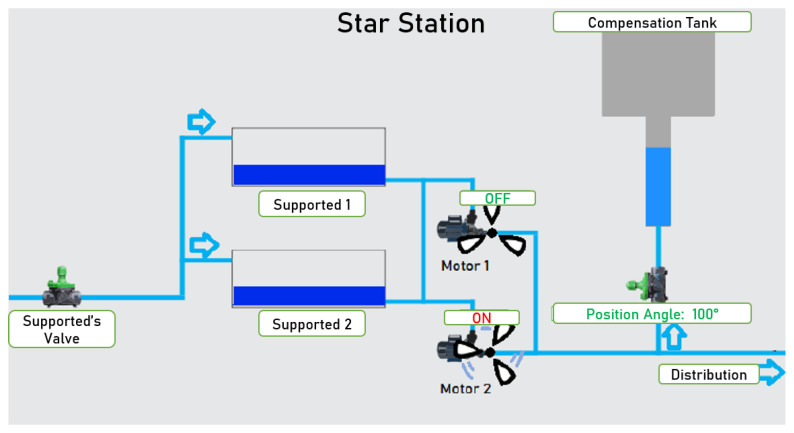
Star Station’s schematic. Source: Cagepa, 2021.

To emphasize the process design, a schematic of this system is presented in Figure 4, considering the hydraulic elements present, such as motors, support reservoirs, valves, and the water box, and also showing the paths of the water injected by the pump sets. In this water lift, the water tank is intended to raise the pressure in the network through its quota. Today, it serves as compensation to help the injection of the network at times of higher consumption. Finally, the support valves regulate the amount of water that arrives at this lift.

The support valve controls the amount of water stored in the support reservoirs to be distributed to that consumption zone. The motor-pump sets work alternatively; one day, the first operates, and the next day, the other operates, prolonging the life of the equipment. The motor pump extracts water from the supported reservoirs and pumps it to the compensation tank and the network. The water tank assists in increasing the load applied to the network.

#### Materials

The Star supply network has two monitoring units, with unit #2 being at the tip of the zone of influence. Their pressures are gauged using an ESP8266 and a pressure transducer for each measurement point, located inside establishments with Internet access owned by the company.

**Figure 5:**
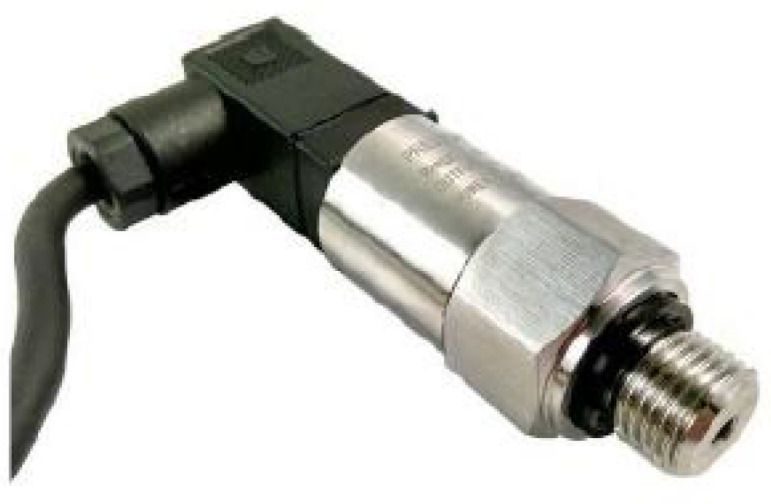
Pressure transducer.

The pressure transducer shown in Figure 5 has a 12-V power supply and provides a 4–20-mA reading signal incompatible with the ESP8266’s 0–3.3-V analog reading. In this regard, a simple circuit was used for signal conditioning that involved a 165-Ω resistor and a capacitor parallel to filter frequencies higher than 60 Hz, promoting an analog readout signal of 0.66–3.3 V.

**Figure 6:**
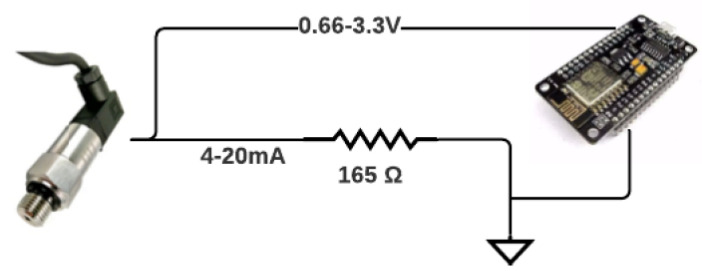
Conditioning circuit for pressure measurement by ESP8266. Source: author.

Figure 6 illustrates the conditioning circuit used to change the current signal given by the hydrostatic sensor to an electrical voltage signal, enabling an analog readout by the ESP8266.

The data read by the ESP8266 were supplied to the supervisory system through the HTTP Receiver protocol every 12 s, totaling 5 measurements per minute. In addition, it was configured for the database to store the average of the measured values every minute. Due to the slow characteristic of the hydraulic system, this sampling rate proved to be satisfactory.

**Figure 7:**
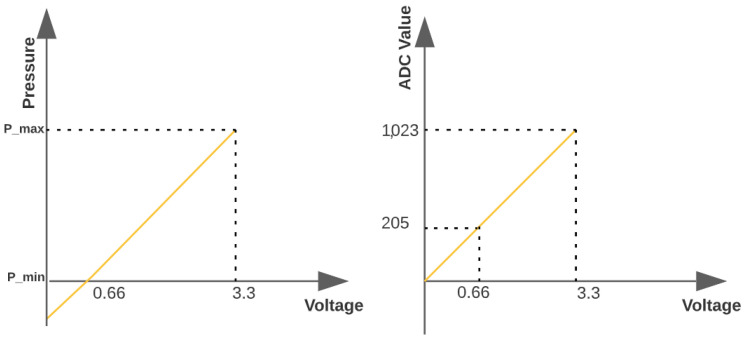
Relationship between measured pressure and converted value for digital form. Source: author.

Furthermore, to convert the analog voltage reading into pressure, we used the fact that the resolution of the ESP8266’s analog-to-digital converter was 10 bits. Thus, a value of 3.3 V corresponded to a value of 1023 (2${}^{10}$− 1). Thus, considering that the hydrostatic distribution sensors measured from 0 to 50 mH${}_{2}$O, the relationship between the measured pressure and the value converted to digital form is shown in Figure 7.

**Figure 8:**
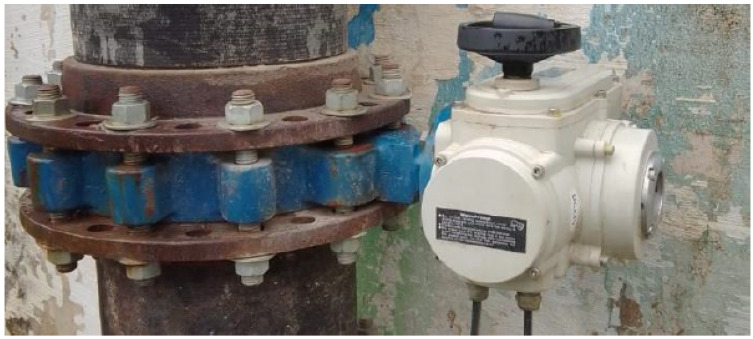
Support reservoir valve. Source: author.

Specifically, the elements present in the WPS of the Star Station included a butterfly-type electric valve. This valve was positioned at the inlet of the supported area. It was an analog-type valve that could be controlled via a 4–20-mA control input, with 4 mA representing a fully closed valve and 20 mA a fully open valve. It was connected to one of the programmable logic controller (PLC) inputs of the S7-1200. In Figure 8, the valve is present in the supported area.

**Figure 9:**
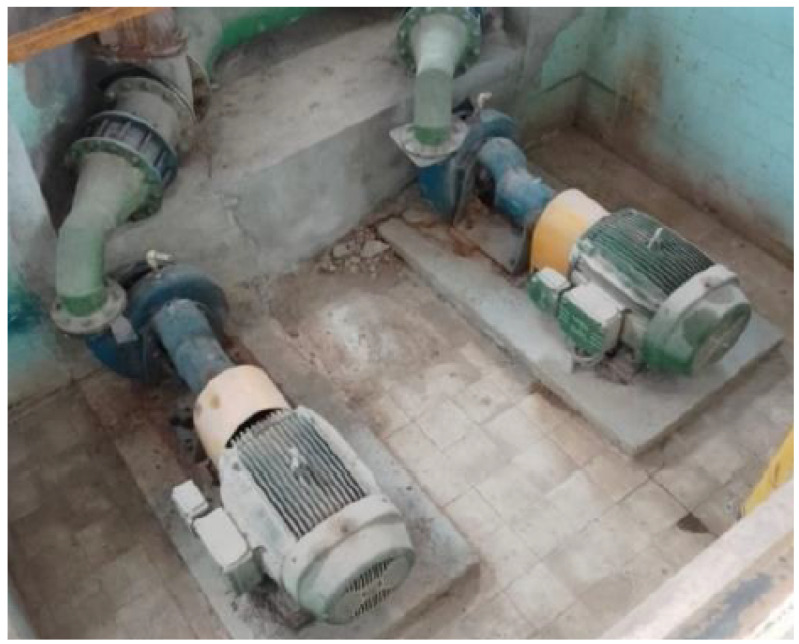
Motor-pump sets. Source: author.

The two sets of motor pumps included motors from the manufacturer WEG with an as-rated power of 45 kW. The pumps were from the manufacturer Ingersoll with a flow rate of 363 m${}^{3}$/h. The sets were positioned in parallel but operated alternately; one day set 1 would operate, and the other day, set 2 would operate. In Figure 9, the motor-pump sets in action and their arrangement in the field are shown.

**Figure 10:**
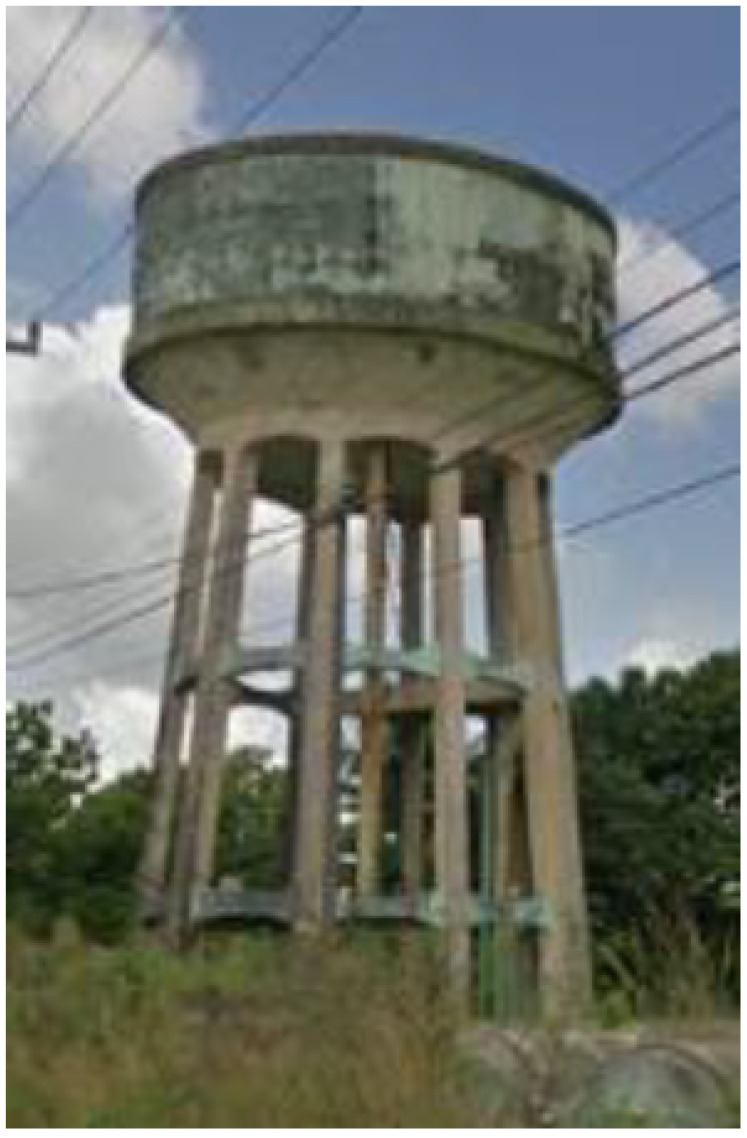
Compensation water tank. Source: author.

For the compensation reservoir, the pumping station’s water tank had a maximum manometric height of 17.5 m. Its supply passed through the same two sets of motor pumps that supplied the population. The water injected from the main pipeline was branched into two lines: one for filling the compensation tank and the other for supplying the population. The water tank can be seen in Figure 10.

**Figure 11:**
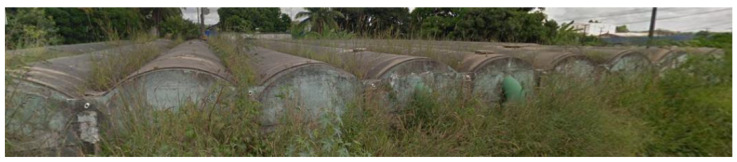
Support reservoirs. Source: Google Maps.

As for the support reservoirs, the WPS of the Star Station has two supports, which are interconnected to each other. The maximum level of the support areas is approximately 3 m. This can be observed in Figure 11.

Regarding the frequency converters, the rotation speed regulation of the pump sets was performed by frequency converters from the manufacturer Danfoss© of the CF202 line. The rotation speed could be controlled by means of a reference input or by sending values via serial communication on the speed control recorder.

**Figure 12:**
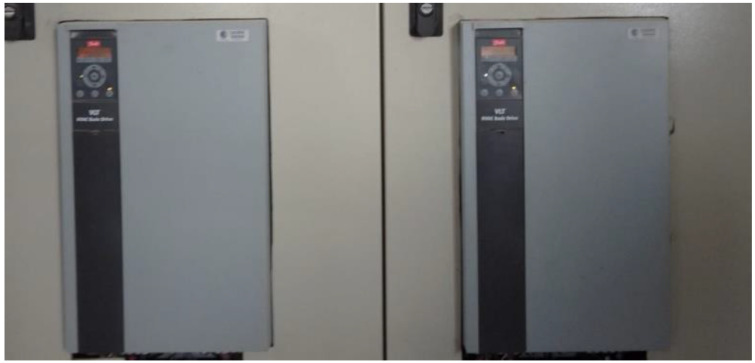
Frequency converters. Source: author.

In this drive, the speed control is accomplished while considering that sending the value 16,384 implies that the motor will rotate at the maximum speed set, and 0 implies it will rotate at the minimum speed. The meaning of these sending values is this way due to the manufacturer’s internal configuration for the Modbus TCP protocol. In Figure 12, one can see the frequency converter inside the control panel.

For communication and control of the lift elements, the Siemens® controller of the S7-1200 DC/DC/DC line was used, which has 8 digital inputs, 6 digital outputs, 1 0–10-V analog input and 2 expansion modules: (1) an RS232/485 module for serial communications, being used for Modbus RTU with the drives, and (2) an expansion module for analog inputs and outputs, containing 4 analog inputs and 2 analog outputs.

**Figure 13:**
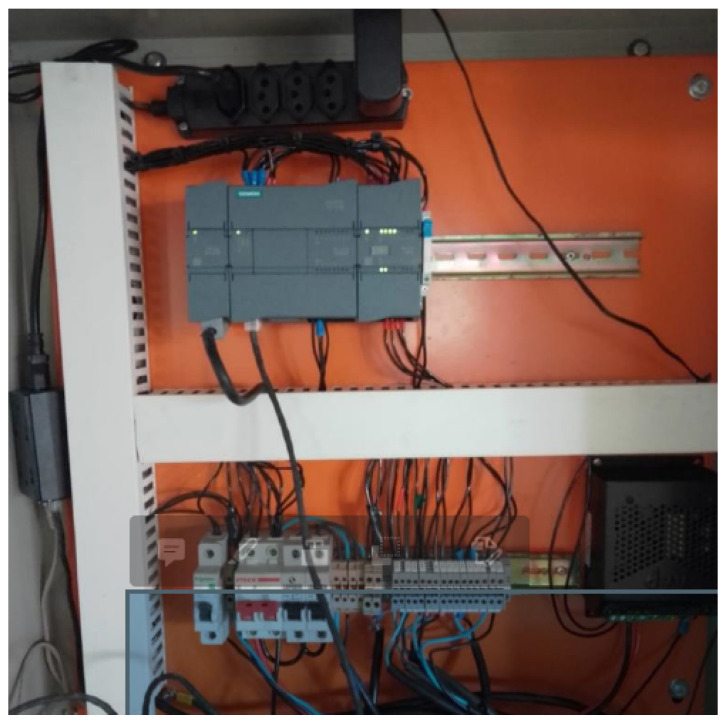
Control panel with Siemens controller. Source: Google Maps.

The installation of the controller in the control panel can be seen in Figure 13.

### 4.2. Methods

This section will discuss in more detail the use of each resource and material used while considering each of the stages. In this paper, the focus is on the reading and acquisition and remote control activities:

1. Reading and acquisition of sensors and actuators.
  - Hydrostatic sensors located in the distribution via ESP8266.
  - Hydrostatic sensors, valves, and frequency converters located in the EEA via PLC S7-1200.
2. Local control.
  - Supported reservoir levels.
3. Remote control.
  - Modeling the system with an ANN using historical data in Python.
  - Development of the fuzzy controller in Python.
  - Validation of the fuzzy control over the ANN model.
  - Real-time data reading from ScadaLTS via the REST API and Python.
  - Send data to ScadaLTS after passing them through the fuzzy controller.

**Figure 14:**
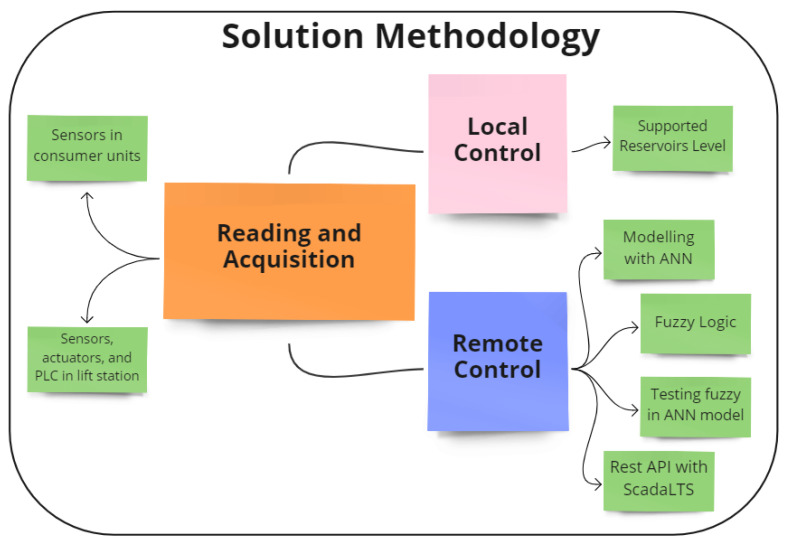
Schematic of the proposed methodology. Source: author, 2022.

In Figure 14, it is possible to observe the proposed methodology in the format of a diagram, relating each of the materials and resources used with the stages developed.

#### 4.2.1. Using the ScadaLTS Features

To be able to consolidate control remotely via Python, it was necessary to use three main ScadaLTS features: the possibility of collecting pressure data stored in the supervisory system database through the REST API, sending the motor rotation speed and pressure data over the network through the Modbus TCP and HTTP receiver protocol, respectively, and the event handler, which can apply the value from one datapoint to another.

Describing the first, the ScadaLTS REST API allows secure integration between an external program and the data stored in the database associated with the supervisor. It is possible to read and write data, access supervisory parameters with simple keywords, and still guarantee security due to the API’s requirement to authenticate access using a valid user name and password. In particular, two GET methods from this API are used:

- /auth/username/password;
  This method allows access to the other API methods by filling in the username and password fields with valid values, ensuring that only people who are registered as users for the supervisory system have access.
- /point_value/getValue/xid.
  This method sends data in JSON format regarding the desired data point from direct database access via xid, a code assigned by ScadaLTS that identifies the desired variable.

Thus, in Python, a routine for reading data from ScadaLTS makes requests for the API every 1 min through the request library. At the same time, the supervisory system stores the desired data in the database.

These requests are made to update the fuzzy controller to the current state of the process with the knowledge of the current inputs (pressure in the distribution) and outputs (rotation speed of the motors).

Up to now, for sending data to the ScadaLTS, three sources have been established from which the data come: the controller located in the WPS, the ESP8266 located in the distribution region, and a computer of the concessionaire’s operational control center, which implements the fuzzy logic that sends the reference speed for the supervisory system.

**Figure 15:**
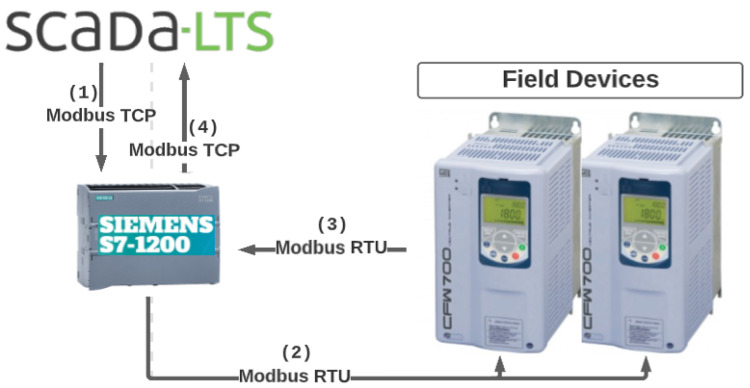
Schematic of the communication between ScadaLTS and frequency converters. Source: Cagepa, 2021.

For the communication of the supervisory system with the frequency converters, the Modbus TCP protocol is applied between the Scada-LTS and the controller so that the controller acts as a gateway in this system, converting the Modbus TCP message received into a Modbus RTU message and making communication with the frequency converters possible. Figure 15 shows the step-by-step schematic of how this communication happens. First, the Scada-LTS makes a request via the Modbus TCP to the controller’s IP. Secondly, the controller decodes the message and converts it to the Modbus RTU, directing the message to the respective inverter or slave. After this, the inverter responds to the controller’s request, finally encoding the message back to the Modbus TCP and answering the controller’s initial request.

For communication with the ESP8266s, the HTTP receiver protocol was used, considering that the only purposes of the boards are to read the output of the hydrostatic pressure sensor and send the measurement value to the ScadaLTS, with no need to receive any commands from the supervisory system.

The HTTP receiver protocol was also used to communicate with the fuzzy controller programmed in Python. Although the Python program needs to take readings and send data through ScadaLTS, the readings are already performed through the API. For simplicity and ease of implementation, it was decided to send the outputs of the fuzzy controller via HTTP and also by the request library.

Finally, for ScadaLTS to send commands to the field equipment, the event handler resource is applied, in which the HTTP variable of the reference speed of the rotation of the motors is applied to the variable of the reference speed of the rotation of the motors in the Modbus TCP, finalizing the final path of the control system proposed by this work.

#### 4.2.2. Process Modeling Based on Artificial Neural Networks

The choice of an artificial neural network as a tool for modeling the hydraulic system was due to one specific factor: we did not have fundamental constructive data of the process, such as the length of the pipes, diameters, and roughness, among others, which excluded the use of simulation software such as Epanet or the realization of mathematical modeling. For this reason, we chose a black box modeling approach, in which the algorithm would learn the relationship between a set of inputs and outputs without understanding the physical process involved.

On the other hand, artificial neural networks are among the most popular and efficient black box modeling techniques. It is possible to find several works in the most varied applications using this technique for modeling purposes. Therefore, the authors chose to apply an ANN of the multi-layer perceptron (MLP) type to model this hydraulic system.

In order to train artificial neural networks, data are required. Therefore, the ScadaLTS report generation resource was used to model the Star supply system, which allowed data to be organized in comma-separated value (CSV) spreadsheets according to the desired time period. These data contained the historical values of the selected data points, which were used to compose the ANN’s training and validation databases.

**Figure 16:**
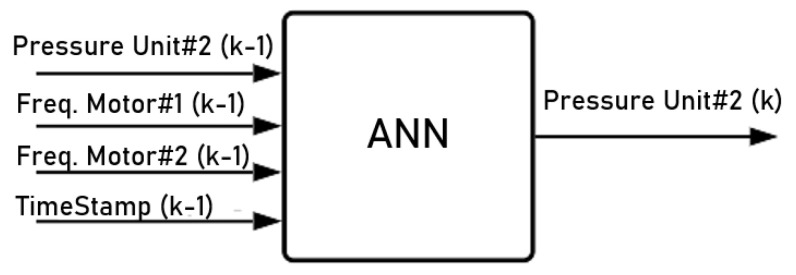
Input–output relationship of the ANN model. Source: author.

For the proposed case study, data from 15 days of operation of the water supply network were collected, having a total of 21,600 samples with a sampling time of 1 min between each one. Figure 16 shows the model’s feedback relationship between the inputs and outputs based on the ANN. In this figure, k is the index of the sampling period, M1 and M2 are the motor pump sets, unit #2 is the pressure measuring point, and TimeStamp is the current minute of the day (e.g., if it is 4:00 a.m., the TimeStamp will be 240 (4*60)).

**Figure 17:**
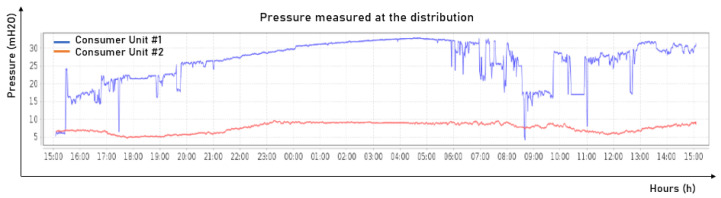
Distribution pressure comparison. Source: Cagepa, 2021.

The pressure control of the lift is based on an incremental speed controller that considers only the error between the set point and the current pressure of consumer unit #2. The decision to use only one consumer unit as the input variable was made after conversations with specialists, since the pressure measured at consumer unit #1 suffered variations due to the installation site of the hydrostatic gauge being in a faucet which was casually opened, causing sudden variations in the pressure measurements. In Figure 17, it is possible to observe the pressure behavior over a day in the two consumer units.

As seen in Figure 17, the pressure measured at consumer unit #1 in blue frequently dropped sharply, with values higher than 5 mH${}_{2}$O occurring many times, making it a very unstable reference. Thus, its use was discarded.

The current logic control that produced the data collected by the report was simple (also created by the author), which caused variations. The rotation frequency of the operating motor pump increased according to the difference between the reference pressure and the measured pressure in discretized increments. Table 1 shows how the current control worked.

#### 4.2.3. Python-Based Fuzzy Control

**Figure 18:**
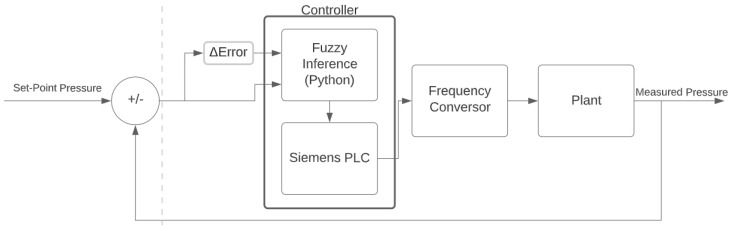
Fuzzy control diagram. Source: author.

To better understand how the fuzzy controller acted in the plant under study, Figure 18 shows a block diagram of the control system. The controller consisted of two devices, one being a computer with fuzzy logic written in Python that sends data to the PLC. Initially, the controller makes an HTTP request to the supervisory system of the measured current pressure to use as input for fuzzy logic the difference between the set point and the current pressure as well as the error variation. The result of the fuzzy inference is transferred to the PLC, which applies the reference speed to the frequency converters acting directly on the hydraulic plant.

The choice of using a fuzzy controller over other traditional controllers, such as proportional–integral–derivative (PID) controllers, was mainly due to the difficult tuning of traditional controllers in industrial processes with complex mathematical modeling and large nonlinearities [28,29,30], as is the case in a hydraulic process.

On the other hand, the controllers were reliable, robust, and resistant to external disturbances to the characteristics of the independent processing of each rule. Moreover, fuzzy rules are simple and easy to understand, and they can be tested individually for each specific situation, being effective for both linear and nonlinear systems [28,29,30,31].

In this sense, the development of fuzzy logic and its rules is a fundamental part of the performance of a fuzzy controller. It is essential to know about the plant and the knowledge of experts to determine, for example, if the rotation speed increment of the motor pump set is small or large enough to change the measured pressure to a value closer to the desired one.

According to [14], the number of terms of the recommended membership functions is between two and seven. In theory, the greater the number of sets, the greater the accuracy of the fuzzy control. However, for values higher than seven, there was no significant improvement [14,28].

In [14,32], these works on pressure control by speed regulation of a frequency converter chose to use two input variables for the fuzzy controller: the error and the difference in the error, with the output being the frequency increment to be performed and having seven sets for each variable and Mandani fuzzy inference systems.

Using these actors’ metodology as reference, the fuzzy logic sets and rules were built, but we changed some set rules and value ranges due to the differences between this case study and the references. The process of the Star water booster station had a slower response time, and the speed updates, due to limitations, were performed every minute (a much longer time than those in the cited references), and there were certain times of the day when the demand of the network was higher than the capacity of the pumping set to supply water to the system.

Considering these assumptions, the input linguistic variables could assume seven possible values: positively big (PB), positively medium (PM), positively small (PS), zero (Z), negatively small (NS), negatively medium (NM), and negatively big (NB). The output linguistic variable could assume the following values: big increment (BI), medium increment (MI), small increment (SI), zero (Z), small decrement (SD), medium decrement (MD), and big decrement (BD).

**Figure 19:**
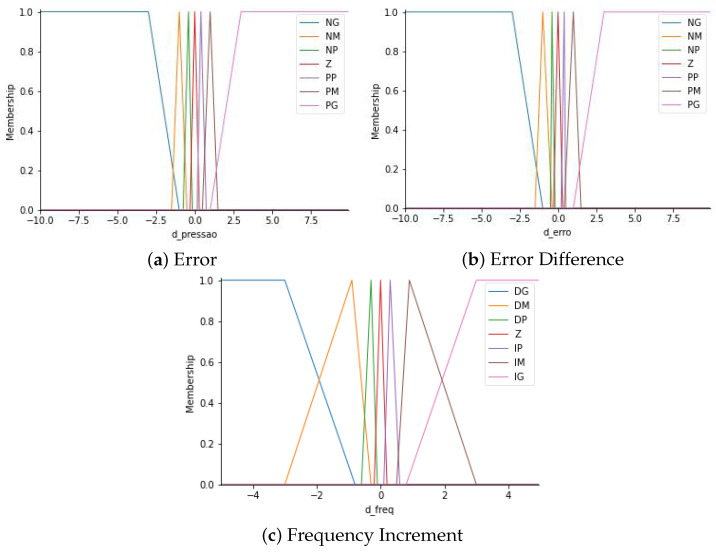
Fuzzy sets. Source: author.

In Figure 19, the constructed fuzzy sets of each linguistic variable of the error, the error difference, and the frequency increment, respectively, can be seen, using as a membership function the triangular function.

**Figure 20:**
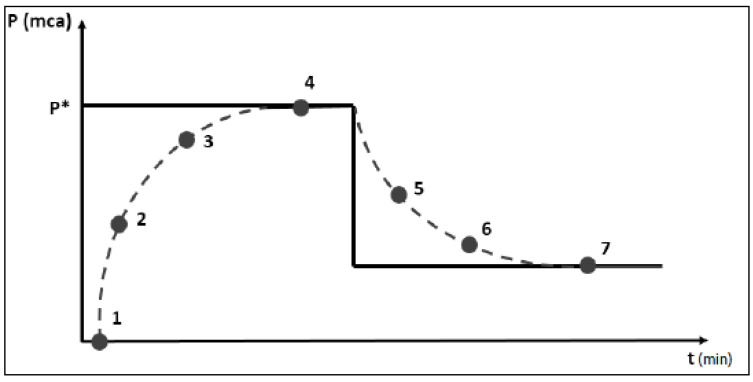
Desired behavior of the fuzzy control. Source: [32], adapted.

The interaction between these sets can be observed through the fuzzy rules developed. The main idea for creating the rules was the frequency’s increment or decrement being found according to the error and the error difference so that the increments were large when the difference between the set point and the measured pressure was high. In contrast, the increment was also smoothed as the error difference was considered high, since a high error difference indicates if the pressure correction is going too fast. In Figure 20, the desired behavior is illustrated, where P* is the set-point.

Table 2 presents the fuzzy rules developed for thinking in this desired behavior. Only “and” logic was used for the interaction between the sets. For example, the frequency variation should be SD for the NB error and NG error difference.

**Figure 21:**
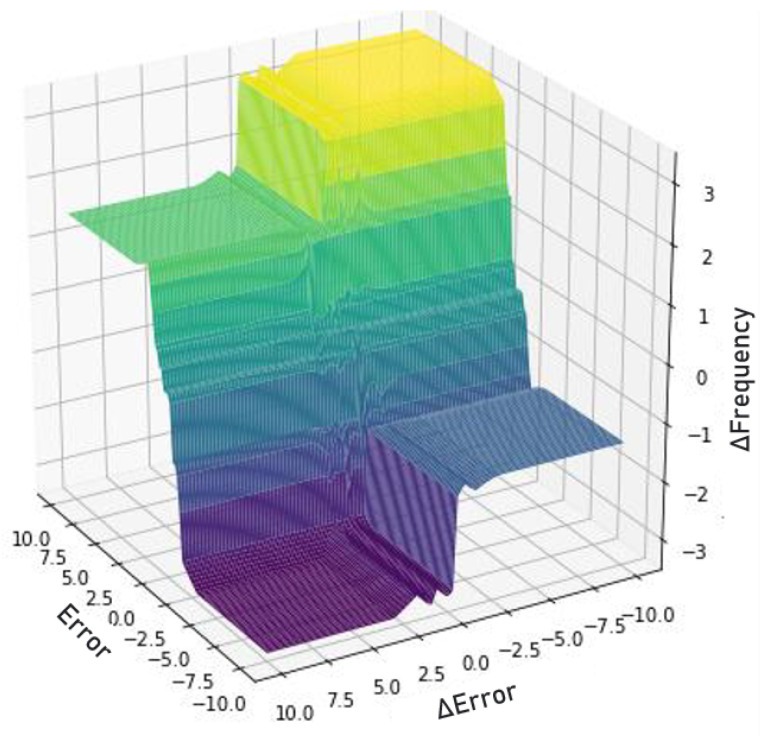
Generated fuzzy surface. Source: author.

According to the literature, a good indication that the interaction between the rules and fuzzy sets is adequate is to observe the relation surface between the inputs and outputs for checking the smoothness of the changes in the output values for different input values. In Figure 21, we can observe the generated surface.

All the development of fuzzy logic was performed using the Python library skfuzzy, which has support for various types of membership functions such as sigmoid, triangular, and trapezoidal functions, with the methods of defuzzyfication as the center of gravity (which was used for the case study) and the average of the maximum values.

**Figure 22:**
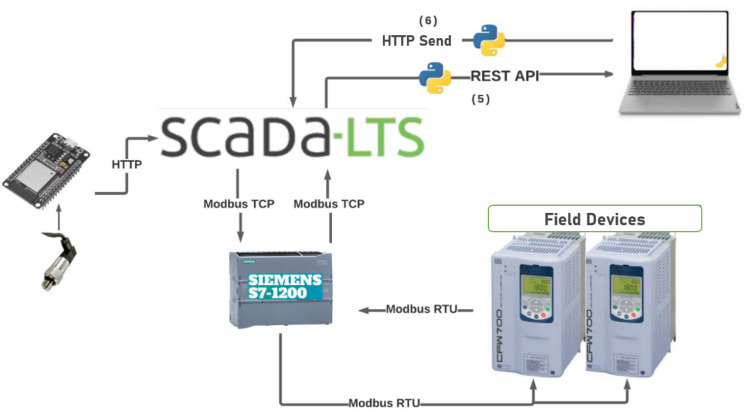
Architecture of the proposed methodology. Source: author.

To summarize the entire methodology proposed, Figure 22 is presented. The final architecture shows control as the whole chain, including measurements of hydrostatic sensors in the field until sending the actuator signal generated by fuzzy logic.

## 5. Results and Discussion

This section will present and discuss the results achieved by the proposed solution, initially comparing the control performance under a model of the Star Station created by an ANN with the current control and then the effect of the control on the real Star WPS.

The following analysis will be presented: performance of the neural network model based on the mean squared error (MSE) and mean absolute percentage error (MAPE) error metrics, the shading test, which will simulate the performance of fuzzy control with real-time data but without acting on field equipment to see if the fuzzy logic has a consistent performance, and finallythe performance of fuzzy control applied over 2 days in the plant, evaluated mainly from the energy point of view.

### 5.1. Analysis of the Results for the ANN Model

After creating the model through ANN modeling with the collected data, the fuzzy controller parameters were adjusted and evaluated in the pressure control of consumer unit #2 to improve the increments and decrements of the frequency rotation frequency converter to operate appropriately.

To evaluate the fuzzy logic’s performance, the same restrictions of the real plant were used: a maximum rotation frequency equal to 62.5 Hz, minimum rotation frequency equal to 43 Hz, and set point pressure equal to 9 mH_2_O, which is 1 mH_2_O less than the minimum preestablished by the ABNT NBR 12,218 standard, the minimum determined by Brazilian law.

Therefore, for all model tests developed, the performance of the fuzzy controller was analyzed under different data conditions with different sets of inputs and outputs, varying the number of neurons per layer, the activation functions used (SeLu and ReLu), and the optimizer (Adam and Nadam).

In this context, the modeling that obtained the best performance had four variables: the pressure in consumer unit #2, the rotation frequencies of the two motors, and the level of compensation. All of them had a sampling rate of one sample per minute. The focus of the work was not to choose the best machine learning model to model the hydraulic network but to create a general solution with fuzzy control, so the technique chosen was a multi-layer perceptron (MLP) neural network. Thus, the best-performing model had the following parameters:

- Two hidden layers with 30 and 20 neurons each;
- Four inputs, namely the rotation frequency of motor 1, rotation frequency of motor 2, pressure of consumer unit #2, and the time of day;
- One output, specifically the pressure from consumer unit #2;
- The ReLu activation function;
- The Nadam optimizer;
- Five thousand epochs.

The model validation was performed using the previously separated data’s two categories: the training data used to create the model and the validation data used to evaluate the model performance.

Table 3, therefore, shows the performance obtained by the model while considering the mean square error and the mean absolute percentage error as parameters.

**Figure 23:**
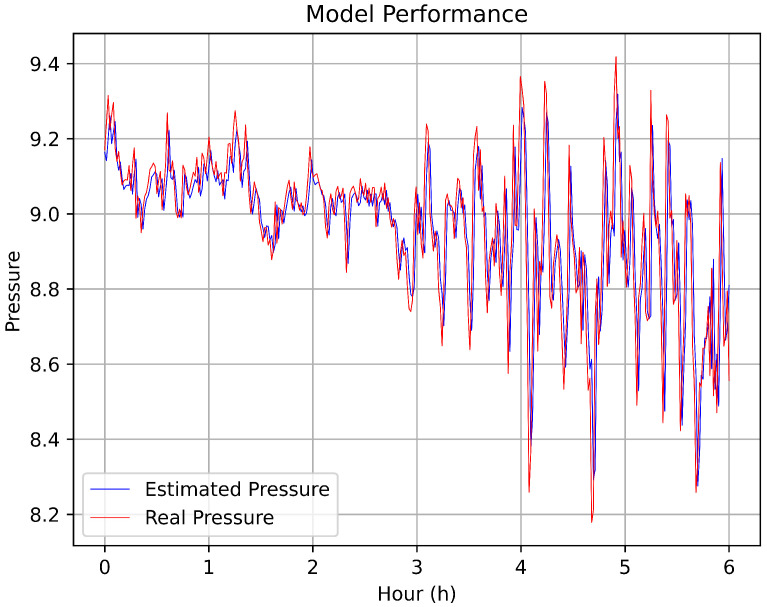
Analysis of the current pressure and that inferred by the neural network for the validation data. Source: author.

Figure 23 shows a graph comparing the actual output values of the pressure at consumer unit #2 and the output values predicted by the ANN, considering the first 6 h of any given test day.

**Figure 24:**
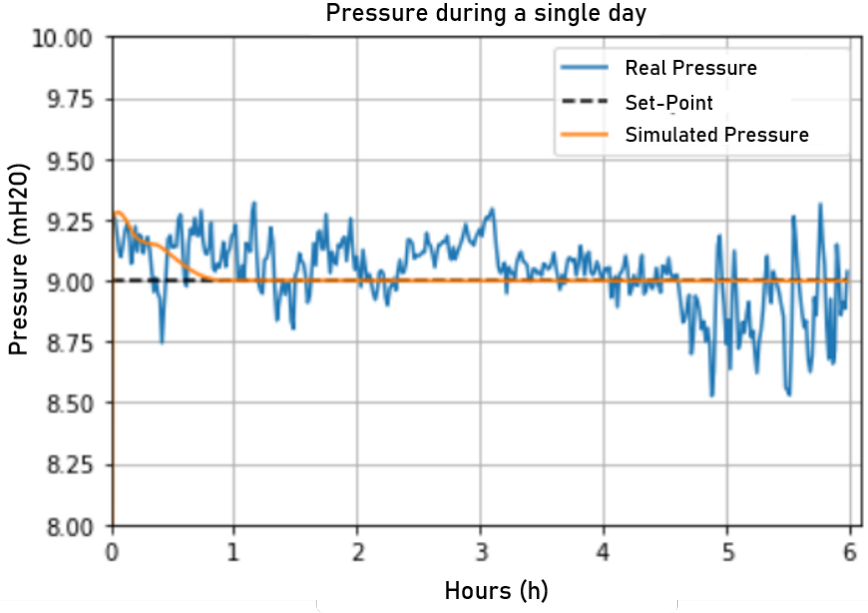
Pressure in the simulated and actual fuzzy networks. Source: author.

To evaluate the simulation performance, a comparison was made between the actual network behavior using the current control and the network behavior during the simulation controlled by the fuzzy controller. Thus, for initialization, the simulation used as input the actual values at minute zero collected from the ScadaLTS system such that the other minutes until 6:00 a.m. were given by the ANN and the fuzzy logic. In Figure 24, a comparison between the simulated and the actual network pressure is presented. The fuzzy control presented more stability than the actual control, showing fewer changes and pressure variations.

**Figure 25:**
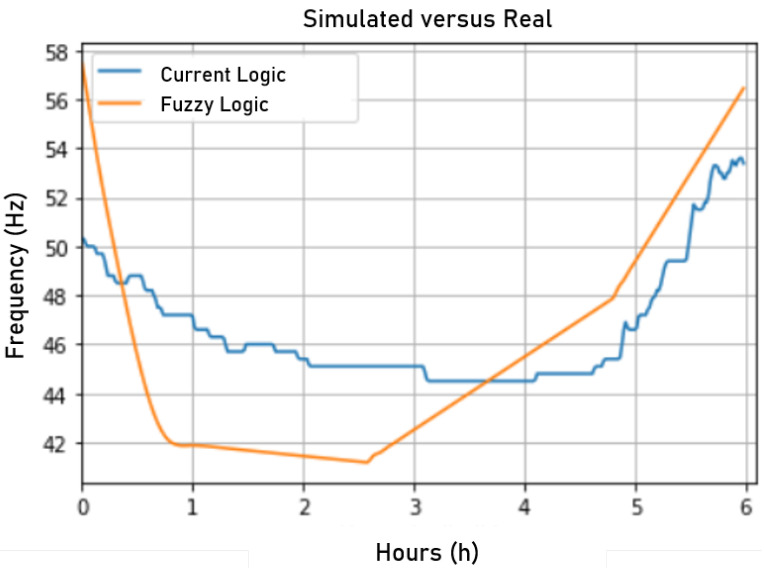
Comparison between simulated and actual fuzzy rotational frequencies. Source: author.

In Figure 25, a comparison between the rotation frequency resulting from fuzzy logic and the rotation frequency resulting from the current control is presented. Interestingly, both frequencies had some correlation. However, the fuzzy control proved to be more stable in controlling the pressure. This lower rotation frequency had the impact of lower energy consumption, making the system more efficient during operation.

### 5.2. Analysis of the Results for the Star WPS

Since the development of the fuzzy control logic was intended to be introduced in a real hydraulic system of water distribution, the care for this should be doubled, and there is no room for misunderstanding or excessive empiricism, which can be detrimental to the whole service. This care was the main reason for building a model for fuzzy logic validation.

Thus, further validation of fuzzy logic was performed before applying the control to the process. To compare the fuzzy control’s coherence with a successful control logic in terms of consistency, a shadowing test was performed. This test worked as follows. Both control logics were monitored and recorded for a certain period, but only the prevailing logic sent the speed reference values to the drives, with the fuzzy logic only in shading mode. The main purpose of this test was to check the consistency between both logics.

Since there was a positive sign in the simulations, it was possible to apply fuzzy logic more safely in the field. Thus, we reached the final part of the work, where fuzzy logic was applied to the frequency converters, analyzing the behavior of the hydraulic network and the rotation speeds of the pumping sets when subjected to fuzzy logic. A performed comparison between the two control logics considered the stability in the grid pressure, the energy difference between the controls, and the frequency variation of the pumping sets as performance parameters.

#### 5.2.1. Shading Test

**Figure 26:**
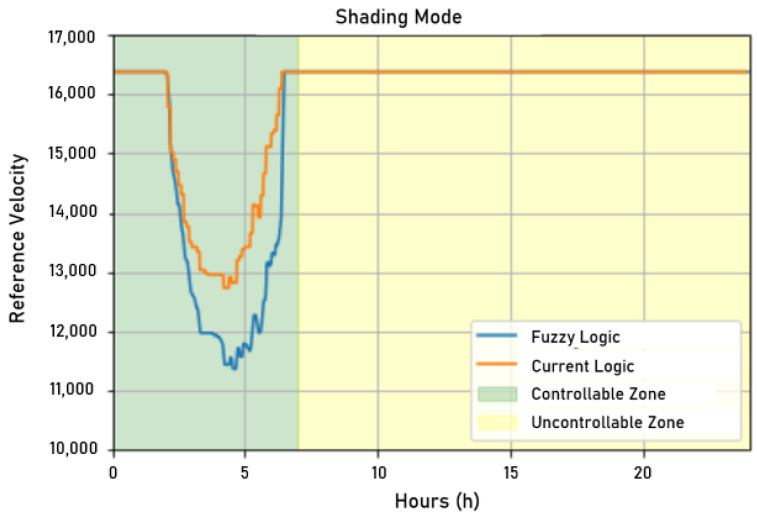
Shading test for fuzzy control. Source: author.

After sending the values processed by the fuzzy controller developed in Python to the supervisory system, it was possible to compare the actuation signals of the current control written in a target variable of ScadaLTS and the fuzzy controller. As shown in Figure 26, considering a visual inspection of the actuation signals, it was observed that the fuzzy controller followed the current controller but acted, theoretically, in a more economical way, since the reference speed sent was lower than that in the current controller. It is worth mentioning again that the system with a single motor in operation could not supply enough water at all times of the day due to peak hour consumption being higher than the supply capacity of the motor pump set. In the graph in Figure 26, the non-controllable zone starts when the reference speed saturates.

#### 5.2.2. Application in a Real Water Network

Once the shading test was over, the fuzzy controller was finally applied to a hydraulic network. To analyze the performance of the proposed solution, this controller was applied to the network to command the control actions of the frequency converters for 2 days and observe the benefits for the consumers and the water distribution company.

Fuzzy control, therefore, was applied during this period, having the most critical period during the early morning, when consumption drops sharply, and the rotation frequency of the motors needed to be precisely controlled to avoid overpressurizing of the network. Thus, the following analyses will focus on this specific time window.

**Figure 27:**
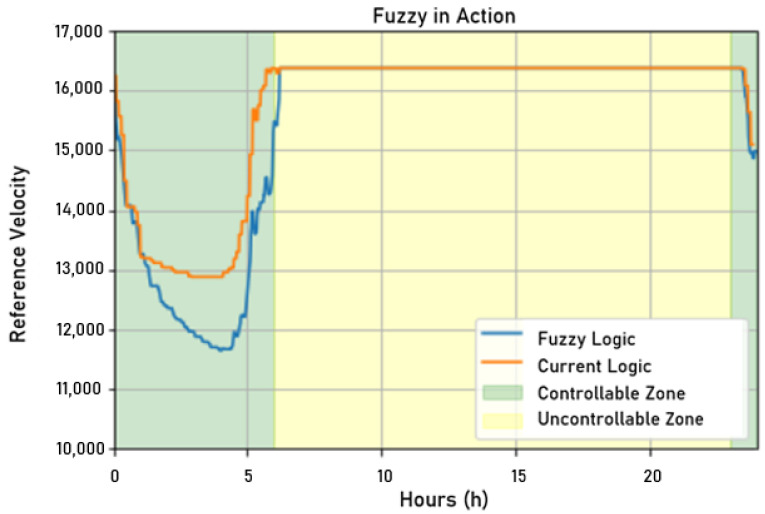
Frequency measured on the first day when applying fuzzy control. Source: author.

In Figure 27, one can observe the relationship between the fuzzy controller’s actuation signals and the current controller, but here, the fuzzy controller commands the control actions. As in the shading test, the fuzzy controller followed the same trend of being more economical than the controller developed with a target variable from ScadaLTS, sending a lower reference speed.

**Figure 28:**
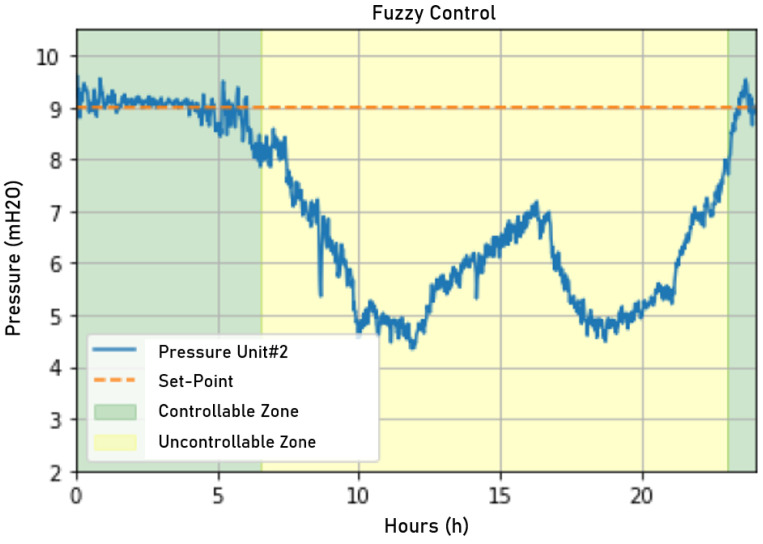
Pressure measured on the first day when applying fuzzy control. Source: author.

The fuzzy controller actuation signal resulted in the network behavior observable in Figure 28. It can be seen that the fuzzy controller acted more stably, with a minor error in the permanent regime, than the incumbent control. However, the resulting controller overvaluation was approximately 6.78%, a small increase over the incumbent controller.

From an economic point of view, the acting power of the motors when controlled by the fuzzy controller was also collected, and we estimated the acting power of the motors (through the actuation signal) if they were on the current control logic to analyze the daily energy difference between both forms of control.

The control signal that started from the current controller had estimated energy spent of 954.40 kWh in a day, while the calculated energy of the fuzzy control was approximately 926.79 kWh in a day, representing savings of just over 27.6 kWh daily.

This set of performances made day 1 of applying the fuzzy control logic very satisfactory, providing benefits at the control and financial levels for the utility.

**Figure 29:**
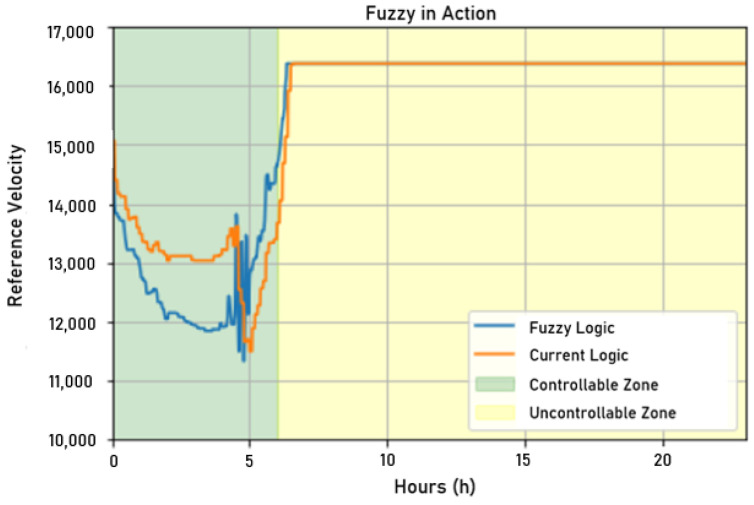
Frequency measured on the second day applying fuzzy control. Source: author.

In Figure 29, there is a comparison between the control actuation signals during the second day of fuzzy controller operation. This time, an unwanted behavior caused a certain degree of instability in the network pressure control. Between 4:00 a.m. and 05:00 a.m., abrupt variations in the actuation signals for both the current control and the fuzzy control can be observed.

The certainty about the origin of this abrupt variation is unknown. However, it is assumed that this was due to the following factor. In a few moments, the interaction between Python and the API with ScadaLTS failed, resulting in delays in request times between the supervisory and fuzzy controllers. This delay may have made the fuzzy controller perceive a very high pressure variation, generating abrupt actuation signals that reflected the pressure applied to the network. However, this failure can be fixed by improving the parameters of the fuzzy sets and rules, giving more robustness to this situation.

**Figure 30:**
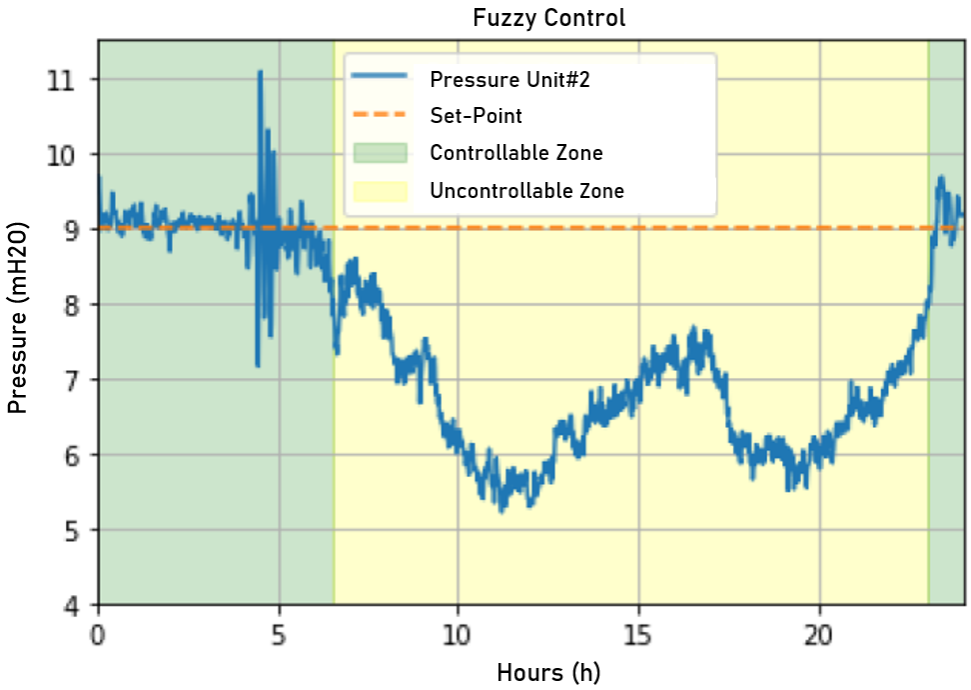
Pressure measured on the second day when applying fuzzy control. Source: author.

This sudden variation caused its impacts on the network, with the control momentarily harming the stability of the pressure control, as can be seen in Figure 30. However, this oscillation was quickly attenuated, showing a fast response from the fuzzy control.

The overvaluation registered after 8:00 p.m. was 7.5%. The instability registered for the pressure control between 4:00 p.m. and 05:00 p.m. can be easily correlated with the sudden variations in the fuzzy controller’s actuation signal, considering that the sudden actuation variations were reflected in a practically instantaneous way by the network pressure.

Thus, it can be seen that there is still a critical bottleneck that can be improved regarding the fuzzy controller parameters. Reducing the sampling rate by one minute and fine-tuning the fuzzy parameters can enhance the control quality.

## 6. Conclusions

The proposed methodology was developed by considering a pressure monitoring and control system based on IoT solutions and artificial intelligence. The solution involved the use of the Modbus TCP protocol to read and write data from field equipment, especially the frequency converters, which are fundamental in varying the rotation speed of the pump sets, the use of ESP8266 boards to send readings from pressure sensors positioned in the distribution, SCADA-LTS as a supervisory system, and Python languages to model the distribution based on an artificial neural network and design the fuzzy controller.

The closed-loop pressure control system was developed by modeling the water supply system using artificial neural networks, designing the fuzzy incremental controller, and developing a supervisory system using open source tools, such as SCADA-LTS. The application of these computational tools allowed for dealing with a nonlinear system with variable demand, allowing the design of classical controllers such as PID complexes.

By implementing the pressure control system in a water supply network, it was possible to establish pressure references in the night hours from 12:00 a.m. to 6:00 a.m., thus eliminating the problem of excess pressure in the pipes due to the drop in water consumption by the population. Then, the lack of a water supply increased due to the rupturing of pipes.

With the advent of technology, extensive use of frequency converters is utilized as a tool associated with energy efficiency actions. In this case, the frequency converters allowed the pump sets to operate with variable rotation speeds and meet pressure control systems’ requirements. In this way, continuous operation with rated frequencies is avoided, increasing energy consumption. Likewise, frequency converters have been the main actors in the replacement of on-off control strategies for motor pump sets.

Future works may explore other machine learning and deep learning techniques to improve the modeling of the system as well as add other relevant input variables. Other communication protocols can also be used, such as MQTT, as an alternative to reading the real-time input data of the fuzzy controller without the dependence on SCADA. Another point is to embed the fuzzy control logic within the industrial controller, providing greater robustness to the control process. Another suggestion would be to use the output of the neural network model as an alternative to the lack of communication with the reference measurement.

## Figures and Tables

**Table 1:** Current control logic.

Error (Set Point and Actual Pressure)	Δf (Hz)
Error > 2	0.9
2 > Error > 1	0.6
1 > Error > 0.3	0.3
Error < −2	−0.9
−2 < Error < −1	−0.6
−1 < Error < −0.3	−0.3

**Table 2:** Fuzzy rules.

Error Difference								
Error		NB	NM	NS	Z	PS	PM	PB
	NG	SD	SD	SD	MD	MD	BD	BD
	NM	Z	SD	MD	MD	MD	MD	MD
	NP	Z	Z	SD	SD	SD	SD	MD
	Z	SI	Z	Z	Z	Z	Z	SD
	PP	MI	MI	SI	Z	SI	Z	Z
	PM	MI	MI	MI	MI	MI	MI	SI
	PG	BI	BI	BI	MI	MI	MI	MI

**Table 3:** Model performance.

Performance	MSE	MAPE (%)
Training	0.0150	0.8393
Validation	0.0203	0.9485

## Data Availability

Not applicable.

## Abbreviations

The following abbreviations are used in this manuscript:

AC	Alternating current
AI	Artificial intelligence
ANN	Artificial neural network
CSV	Comma-separated values
HTTP	HyperText Transfer Protocol
IoT	Internet of Things
PID	Proportional–integral–derivative
PLC	Programmable logic controller
RTU	Remote terminal unit
SCADA	Supervisory, control, and data acquistion
TCP	Transmission control protocol
TWPS	Treated water pumping station
WPS	Water power supply
WDS	Water distribution system
